# Development of a 45K pepper GBTS liquid-phase gene chip and its application in genome-wide association studies

**DOI:** 10.3389/fpls.2024.1405190

**Published:** 2024-06-25

**Authors:** Zixiong Li, Zhiqi Jia, Jisuo Li, Dongmu Kang, Mingxuan Li, Shijie Ma, Qing Cheng, Huolin Shen, Liang Sun

**Affiliations:** ^1^ Beijing Key Laboratory of Growth and Developmental Regulation for Protected Vegetable Crops, Department of Vegetable Science, College of Horticulture, China Agricultural University, Beijing, China; ^2^ Department of Vegetable Science, College of Horticulture, Henan Agricultural University, Zhengzhou, China; ^3^ Beijing Bona Oriental Agricultural Technology Development Co., Ltd, Beijing, China

**Keywords:** pepper, germplasms, GBTS liquid-phase gene chip, polymorphic site, helical-shaped fruit

## Abstract

**Introduction:**

Pepper (*Capsicum* spp.) is a vegetable that is cultivated globally and has undergone extensive domestication, leading to a significant diversification in its agronomic traits. With the advancement of genomics in pepper and the reduction in sequencing costs, the high-throughput detection of single nucleotide polymorphisms (SNPs) and small insertions-deletions (indels) has become increasingly critical for analyzing pepper germplasms and improving breeding programs. As a result, there is a pressing need for a cost-effective, high-throughput, and versatile technique suitable for both foreground and background selection in pepper breeding.

**Methods:**

In the present study, Python-based web scraping scripts were utilized to systematically extract data from published literatures and relevant sequence databases focusing on pepper genomes. Subsequent to data extraction, SNPs and indels were meticulously identified and filtered. This process culminated in the delineation of core polymorphic sites, which were instrumental in the development of specific probes. Following this, comprehensive phenotypic and genotypic analyses were conducted on a diverse collection of 420 pepper germplasms. Concurrently, a genome-wide association study (GWAS) was conducted to elucidate the genetic determinants of helical fruit shape in peppers.

**Results:**

In this study, a 45K pepper Genotyping-By-Target-Sequencing (GBTS) liquid-phase gene chip was developed on the GenoBaits platform. This chip is composed of 45,389 probes, of which 42,535 are derived from core polymorphic sites (CPS) in the background genetic landscape, while 2,854 are associated with foreground agronomic traits, spanning across 43 traits. The CPS probes are spaced at an average interval of 68 Kb. We have assessed the performance of this chip on 420 pepper germplasms, with successful capture of target DNA fragments by 45,387 probes. Furthermore, the probe capture ratio surpassed 70% in 410 of the 420 germplasms tested. Using this chip, we have efficiently genotyped 273 germplasms for spiciness levels and elucidated the genetic relationships among 410 pepper germplasms. Our results allowed for precise clustering of sister lines and *C. chinense* germplasms. In addition, through a GWAS for helical fruit shape, we identified three quantitative trait loci (QTLs): *heli2.1, heli11.1,* and *heli11.2.* Within the *heli11.1* QTL, a gene encoding the tubulin alpha chain was identified, suggesting its potential role in the helical growth pattern of pepper fruits.

**Discussion:**

In summary, the 45K pepper GBTS liquid-phase gene chip offers robust detection of polymorphic sites and is a promising tool for advancing research into pepper germplasm and the breeding of new pepper varieties.

## Introduction

Pepper (*Capsicum* spp.), originating from the northwestern Andes, is a globally cultivated, vital vegetable crop with over 6,000 years of domestication history (Perry et al., 2007). It primarily encompasses five domesticated species: *C. annuum*, *C. frutescens*, *C. chinense*, *C. pubescens*, and *C. baccatum*. These species exhibit diverse traits in fruit morphology, pigmentation, capsaicin levels, plant architecture, flowering patterns, as well as disease and stress resistance (Wu et al., 2019; Cao et al., 2022), forming a rich germplasm resource for breeding new, improved pepper varieties.

Advancements in molecular biology have revolutionized pepper breeding, transitioning from phenotypic selection to molecular marker-assisted selection, and now to comprehensive genome-wide selection. The advent of next-generation sequencing technologies and the sequencing of the pepper genome have uncovered a plethora of single nucleotide polymorphisms (SNPs) and insertions/deletions (indels) (Kim et al., 2014; Qin et al., 2014). These discoveries have enabled the identification of key loci governing agronomic traits as well as the cloning of their associated genes. A substantial number of quantitative trait loci (QTL) concerning fertility (Cheng et al., 2018, 2020), first flower node (Zhang et al., 2018), fruit quality (Nimmakayala et al., 2016, 2021), and disease resistance (Kang et al., 2016) have been reported, setting the stage for development of high-throughput, genome-wide selection techniques.

In modern plant breeding, selection is refined through molecular techniques. Foreground selection employs molecular markers linked to desired agronomic traits to ensure their passage to future generations. Background selection screens for chromosomal regions outside targeted loci to hasten backcrossing and breeding homogeneity. High-throughput methods, such as whole-genome resequencing and gene chip technologies, have predominated in genome-wide selection. Recently, Genotyping by Target Sequencing (GBTS) has emerged as a cost-effective alternative to whole-genome sequencing and offers more flexibility over solid-phase gene chips. GBTS, leveraging second-generation sequencing, captures specific DNA fragments using designed primers or probes, followed by PCR amplification and in-depth sequencing (Samorodnitsky et al., 2015). GBTS can be categorized into GenoPlexs, using multiplex PCR for target amplification, and GenoBaits, employing liquid-phase probe hybridization for selective DNA capture (Guo et al., 2019). GBTS chips are adaptable, compatible with various sequencing platforms, efficient, and offer a simpler analysis process. Their application extends across various crops, such as maize (Guo et al., 2021), soybean (Liu et al., 2020; Yang et al., 2023), cotton (Si et al., 2022), rice (Lee et al., 2022), wheat (Xiang et al., 2023), barley (Nie et al., 2022), peanuts (Lu et al., 2023), tomato (Li et al., 2022), and pepper (Miao et al., 2023), and genetic research areas, facilitating effective germplasm assessment, breeding material selection, and genotypic analysis (Liu et al., 2020; Guo et al., 2021; Lee et al., 2022; Si et al., 2022; Xiang et al., 2023; Yang et al., 2023).

In pepper, to date, two solid-phase gene chips and one liquid-phase chip have been reported. The inaugural 30K Pepper GeneChip®, predating the pepper genome publication, was based on 30,815 Sanger-EST assembled unigenes (30K) and facilitated the development of molecular markers for spicy loci and population structure analysis (Hill et al., 2013). Following the genome release, a 15K SNP array chip was created, utilizing Illumina’s Infinium iSelect technology, which covered 81% of the genome and aided in constructing a high-density genetic map and performing population diversity analysis (Cheng et al., 2016). Despite the abovementioned chips, the progress in liquid-phase GBTS gene chips for peppers lags, with only a 5K chip patent containing 5,984 SNPs (Miao et al., 2023). This gap highlights the need for higher-capacity GBTS chips to enhance the efficiency and cost-effectiveness of marker screening in pepper breeding.

In pepper cultivation, the spiciness, determined by capsaicinoids content, and fruit shape significantly influence market value. Capsaicinoids, primarily capsaicin and dihydrocapsaicin, are amide alkaloids synthesized via the phenylpropanoid and branched-chain fatty acid pathways, involving key genes like *PAL*, *C4H*, *4CL*, and others (Curry et al., 1999; Aluru et al., 2003; Stewart et al., 2005; Abraham-Juárez et al., 2008; Kim et al., 2014; Qin et al., 2014). These compounds activate the TRPV1 heat receptor, eliciting a pain response (Caterina et al., 1997; Ji et al., 2002). QTLs linked to capsaicin levels have been identified across multiple chromosomes (Park et al., 2019; Jang et al., 2021). Consequently, the establishment of a molecular marker system capable of identifying capsaicin content or the type of spiciness would considerably propel the breeding of new pepper varieties. Fruit shape significantly impacts pepper quality and market suitability, with helical or spiral growth as a distinct and desirable feature, particularly in central, northwest, and southwest China where such varieties are increasingly popular. Despite the rising cultivation, knowledge on the genetic control of helical fruit shape is limited. This trait, observed in various plant organs, contributes to climbing, seed dispersal, and photosynthetic efficiency (Schulgasser and Witztum, 2004; Smyth, 2016; Sousa-Baena et al., 2021). Research suggests helical growth is due to cellular microtubule alterations. Genes like *SPIRAL1*, *SPIRAL2*, *SPIRAL3*, (GCP2), *LEFTY1*, *LEFTY2*, *WVD2* and *WDL* in Arabidopsis encode microtubule-related proteins, with mutations causing spiral growth in several plant organs, such as hypocotyl, stem, petiole, petals, and roots (Thitamadee et al., 2000; Yuen et al., 2003; Nakajima et al., 2004; Shoji et al., 2004; Nakamura and Hashimoto, 2009). Similarly, mutations in genes encoding CML24, IQ67, and RHM1 are also linked to helical organ development (Wang et al., 2011; Wu et al., 2011; Saffer et al., 2017). Nevertheless, in pepper, our understanding remains limited regarding the specific loci or genes that govern the development of helical-shaped fruits.

In this study, aiming to advance the pepper breeding industry with a more adaptable selection platform and to elucidate the genetic determinants responsible for the development of helical-shaped fruits, we have developed a 45K pepper GBTS liquid-phase gene chip. This cutting-edge tool, crafted using GenoBaits technology, was informed by an extensive review of 89 pepper-related scientific articles and patents from the past two decades. This chip was meticulously designed with probes which were developed from 45,389 core polymorphic sites, strategically dispersed over 12 chromosomes at an average separation of 68 kb. Utilizing this chip, we have proficiently genotyped 420 pepper germplasms, categorizing them by spiciness level and delineating their population structure—a testament to the chip’s exceptional DNA fragment capture efficiency. In a landmark application, the chip facilitated a GWAS that unearthed 3 quantitative trait loci (QTL) and one candidate gene associated with the helical fruit shape in peppers. These breakthroughs suggest that the gene chip is not only a valuable asset for future pepper breeding but also a pivotal resource for identifying key loci that regulate agronomic traits.

## Materials and methods

### Acquisition of pepper polymorphic sites data

Initially, Python-based web scraping scripts were employed to extract data from the internet, specifically targeting published literature and corresponding sequence databases pertaining to pepper genomes. The search included terms such as agronomic traits, gene positioning, gene cloning, SNPs, indels, GWAS, Zunla1, and CM334. The retrieved sequence data were then aligned to the reference pepper genome, designated as CA59 (Liao et al., 2022), utilizing the BWA MEM algorithm (Li and Durbin, 2009). Following alignment, the sequencing data were organized and deduplicated using Samtools (Danecek et al., 2021). Polymorphic sites were identified with the aid of BCFtools (Danecek et al., 2021). Polymorphic sites discerned through alternate reference genomes were reconciled to the CA59 genome using the BLAST tool version 2.14, available at NCBI BLAST (https://blast.ncbi.nlm.nih.gov/Blast.cgi). For instances where only primer information was available without corresponding sequence data, BLAST version 2.14 was utilized to ascertain the precise loci within the CA59 genome. The final step entailed pinpointing the locations of the genome sequences from various pepper materials within the CA59 reference genome.

### SNP and small indel filtering

A variant call format (VCF) file was generated incorporating the variant information from the pepper germplasm obtained in the preceding step. Polymorphic sites were then filtered using Plink (Purcell et al., 2007) and VCFtools (Danecek et al., 2011) according to the following criteria: (1) a minimum sequencing depth of 10X; (2) a minor allele frequency (MAF) greater than 0.05; (3) a missing data ratio of less than 20%; (4) a heterozygosity ratio of less than 5%; (6) linkage disequilibrium (LD) of less than 0.4; (6) Probe regions must be free of simple sequence repeats (SSRs) and ambiguous nucleotides (denoted as ‘N’); (7) variants other than A/T and C/G were given priority. All SNPs and small indels satisfying these conditions were subsequently identified as core polymorphic sites for the development of gene chip probes.

### Development of gene chip probes

The cornerstone of liquid-phase gene chip technology is the employment of probes that are complementary to target sequences for their capture. In this research, polymorphic sites linked to agronomic traits were singled out as foreground sites from the aggregate of core polymorphic sites, while the remainder were designated as background sites. To select foreground sites for probe development, literature gathered via Python web scraping scripts was examined to pinpoint polymorphic sites associated with key agronomic traits, including capsaicin content, fruit color, and disease resistance, among others. Priority was assigned to those polymorphic sites with empirically validated functions when selecting candidate foreground sites. Further, polymorphic sites situated within major-effect quantitative trait loci (QTLs) that have been meticulously mapped in the literature were also considered as candidates. Moreover, candidate foreground sites from existing gene chips were taken into account for the creation of foreground probes on this new chip. The aforementioned polymorphic sites constituted the foundation for gene chip development and will be employed in the future for the generation of foreground probes. Additionally, the MEME platform (http://meme-suite.org/tools/meme) was utilized to analyze genes linked with core polymorphic sites to further ascertain their potential functions. Once foreground sites were identified, they were located on the CA59 pepper reference genome. The distance between contiguous foreground sites was computed, and extensive vacant regions with intervals of 70 kb or more were recognized. To populate these extensive vacant intervals, polymorphic site data from 287 pepper germplasms (Wu et al., 2019) were employed, ensuring a balanced distribution of core polymorphic sites throughout the 12 pepper chromosomes.

The probes for the GenoPlexs® gene chip were designed to be 110 bp in length, with a GC content ranging from 30% to 80%. The chosen regions excluded simple repetitive sequences and blank regions (Guo et al., 2021). A/T or C/G type SNPs were deliberately avoided, as other SNP types necessitate only one probe for hybridization, whereas the former require two, thus escalating the cost of chip design. Further filtering based on MAF values, NA (missing data) values, and heterozygosity (Het) values was conducted using Plink (Purcell et al., 2007) and VCFtools (Danecek et al., 2011). Subsequently, the uniqueness of each core polymorphic site within the genome was verified by executing a single-copy test on the candidate segments, which included sequences 200 bp upstream and downstream of each polymorphic site, using BLAST v2.14 (https://blast.ncbi.nlm.nih.gov/Blast.cgi). The development of gene chip probes was finalized based on these vetted candidate segments.

### Plant materials and genotyping

In this study, we utilized 420 pepper germplasm samples to validate the performance of the pepper GBTS gene chip. These germplasm resources encompassed *C. annuum*, *C. chinense*, and sister lines sharing identical genetic backgrounds (**Supplementary Table S1**). The pepper germplasms were cultivated in plastic greenhouses at the Shangzhuang Experimental Station of the China Agricultural University. Cotyledons were harvested for sampling when the plants were at the four-leaf stage. Genomic DNA was extracted using the CTAB method (Saghai-Maroof et al., 1984). DNA concentration was measured with a NanoDrop instrument, and DNA quality was verified through agarose gel electrophoresis.

According to the technical manual, 200 ng of genomic DNA (gDNA) from each pepper germplasm was hybridized onto the chip. Gene chip sequencing data obtained were aligned to the pepper reference genome Ca59 using the BWA MEM algorithm (Li and Durbin, 2009). To avoid increased error rates in genetic analyses that could lead to incorrect conclusions, variants with excessive amounts, low minor allele frequency (MAF) values, high missing data (NA) values, and high heterozygosity (Het) values were filtered out from the original sequencing data. The Plink software (Purcell et al., 2007) was employed to filter background markers based on the criteria of: (1) MAF less than 0.05; (2) missing rate greater than 0.1; (3) heterozygosity ratio greater than 0.8. Following the sequencing data quality control, DNAsp (Rozas et al., 2017) was used to identify haplotype regions significantly associated with traits. Custom Python scripts were employed to statistically analyze and visualize the detection effects of foreground loci probes. Excel 2016 was utilized to compute the average capture rate of each probe for each germplasm. Finally, the ANNOVAR tool (Wang et al., 2010) was used for the annotation of gene regions targeted by each probe.

### Genotyping of spiciness of pepper germplasms

The spiciness level of the fruit was determined using the taste test method. For each of the 6-8 robustly growing plants, 3-4 fully developed fruits from equivalent positions were selected at the mature green stage for spiciness assessment. The spiciness of pepper fruits was categorized into four distinct types: sweet, mildly spicy, moderately spicy, and spicy. “Sweet” denotes a flavor profile that is devoid of any spiciness; “mildly spicy” describes a sweet base flavor with a subtle hint of spiciness; “moderately spicy” signifies a spiciness level that is perceptible but not overpowering; “spicy” designates a high level of spiciness. To accommodate individual variance in spiciness tolerance, the sensory evaluation was performed by a diverse group of 10-15 individuals differing in gender, age, geographic backgrounds, and dietary preferences. The tasting began with the fruits classified as “sweet” and “mildly spicy,” followed by those identified as “moderately spicy” and “spicy.” The final classification of the samples was based on the consolidated average of the taste test results.

In addition, for the classification of the helical-shaped fruit phenotype, 3-4 well-developed fruits from consistent positions were selected from each of the 6-8 healthy plants at the turning stage. For the purposes of statistical analysis, any fruit exhibiting signs of helical growth was labeled as helical. In contrast, fruits without helical characteristics were designated as non-helical.

### Genome-wide association study

High-quality single nucleotide polymorphisms (SNPs) and small insertions-deletions (indels) derived from the pepper gene chip were utilized for genome-wide association studies (GWAS). To impute missing gene loci in the sequencing data, the Beagle software (Browning et al., 2018) was employed, referencing the pepper genome sequences. Subsequently, the imputed.vcf files were converted to.ped and.map (or.bed) formats using the PLINK software (Purcell et al., 2007) in preparation for further analyses. Population structure was investigated using ADMIXTURE software (v1.3) (Alexander et al., 2009). We initially set K values ranging from 1 to 10 and performed clustering analyses to delineate genetic populations. The optimal K was identified based on the minimum cross-validation error (CV error). For phylogenetic tree construction, genetic distances at polymorphic sites were calculated utilizing MUSCLE software (Edgar, 2004), which were then optimized with TrimAl software (Capella-Gutiérrez et al., 2009). The ML phylogenetic trees were generated using IQ-TREE software (Minh et al., 2020). The resulting trees were visualized and refined on the Evolview website (https://evolgenius.info//evolview-v2). Kinship among the samples was assessed using TASSEL software (Bradbury et al., 2007), which facilitated the correction for relatedness in the GWAS. The population structure and kinship analyses were graphically represented with plots produced in RStudio. Following rigorous quality control and alignment of sequencing data, the GAPIT package (Lipka et al., 2012) and TASSEL 5.0 were utilized for the association analyses. To account for multiple testing, the Bonferroni correction was applied, setting a stringent threshold for identifying loci significantly associated with the traits of interest. Data visualization, including the creation of Manhattan and QQ plots, was performed using the ‘CMplot’ package in R.

## Results

### Development of the pepper GBTS liquid-phase gene chip

**Figure 1:**
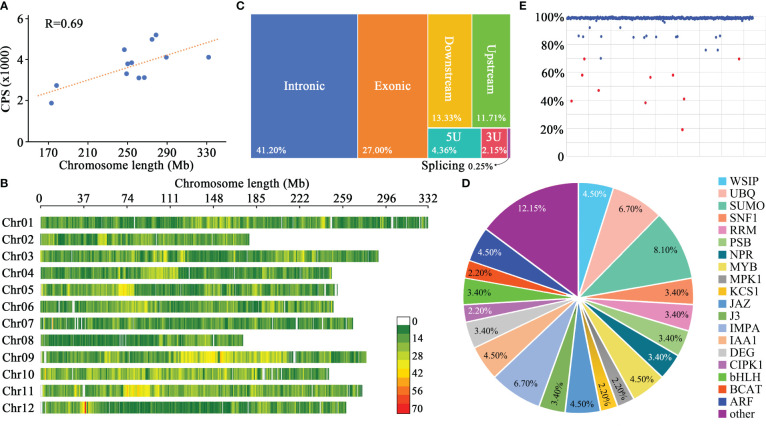
Fundamental parameters and probe capture efficiency of the 45K gene chip. **(A)** Chromosome length and count of core polymorphic sites (CPS). **(B)** Spatial arrangement of CPS across pepper chromosomes, with the color scale indicating CPS density per 1 Mb segment. **(C)** Proximity analysis of the 6241 CPS relative to annotated genes. **(D)** Detailed annotations for genes adjacent to CPS. **(E)** Capture efficiency of the 420 pepper germplasms.

Using Python-based web scraping techniques, we systematically collated data from 89 pertinent publications, as detailed in **Supplementary Table S2**. Central to our analysis were three pivotal studies: our team’s previously conducted genome-wide association study (GWAS) on 287 pepper germplasms (Wu et al., 2019); the solid-phase 30K pepper chip devised by South China Agricultural University researchers (Cheng et al., 2016); and a comprehensive examination of phenotypic variations in fruit morphology across 244 C*. chinense* germplasms by West Virginia State University researchers (Nimmakayala et al., 2021). This comprehensive literature survey yielded sequence variation data for 2,361 pepper germplasms encompassing a diverse *Capsicum* genus – *C. annuum*, *C. frutescens*, *C. chinense*, *C. pubescens*, and *C. baccatum*. A total of 45,389 core polymorphic sites met our selection criteria, comprising 1,869 foreground sites, 985 functional segments, and 42,535 background sites. The number of core polymorphic sites on each chromosome ranged from 1,857 to 5,198, with an average of 3,720.5 (**Figure 1A**), and a strong correlation between the number of core polymorphic sites per chromosome and chromosome length was observed (**Figure 1A**), indicating that the core polymorphic sites in this chip are evenly distributed across the genome. Furthermore, the average spacing between two adjacent sites ranges from 55 kb to 70 kb, with an average interval of 68 kb, further indicating an even distribution of the core polymorphic sites across chromosomes and a good genome-wide detection capability (**Figure 1B**).

Digging deeper, we found that out of the 45,389 core polymorphic sites, 6,241 were in proximity to 5,410 annotated genes. Breaking down their genomic positions, 2,571 sites were intronic, 1,685 were exonic, 1,563 were situated within 3 kb proximal to gene boundaries, 406 were present in untranslated regions (5’ UTRs or 3’ UTRs), and 16 had implications for alternative splicing (**Figure 1C**). The associated genes encompassed transcription factors, enzymes, and signal transducers etc., which implicated in the development of plant organs such as roots, stems, and leaves, as well as diseases and plant hormones pathways (**Figure 1D**).

In this study, foreground sites were identified based on previously documented correlations between polymorphisms and a spectrum of agronomic traits in the literatures. These sites were linked to 43 agronomic traits, comprising both resistance characteristics—such as to powdery mildew, root-knot nematodes, cucumber mosaic virus, tobacco mosaic virus, pepper phytophthora, aphids, anthracnose, potato virus Y, bacterial wilt, and thrips—and various fruit attributes, including chlorophyll concentration, alpha-glucosidase inhibition potential, dry matter content, capsaicin levels, flavonoid content, pigmentation, shape, shoulder morphology, surface luster, flesh thickness, growth orientation, attachment site, weight, and locule number. Additionally, polymorphic sites were associated with leaf pigmentation, the node of initial flowering, propensity for multiple flowers, corolla and anther coloration, and the length of inflorescence stems (**Table 1**). Notably, the largest numbers of polymorphic sites were found in association with traits such as pepper branching type (304 sites), the multi-flowered characteristic (177 sites), and the node of first flowering (167 sites), as enumerated in **Table 1**.

**Table 1:** Number of polymorphic sites associated with key agronomic traits in pepper.

Traits related to foreground sits	Number of loci	Traits related to foreground sits	Number of loci
Resistance to powdery mildew	129	Leaf color	1
Resistance to root knot nematodes	6	Fruit shoulder shape	6
Resistance to cucumber mosaic virus	140	Fruit surface luster	1
Resistance to tobacco mosaic virus	3	Pericarp thick	12
Resistance to pepper blight	27	Fruit orientation	46
Resistance to aphids	1	Fruit position	25
Resistance to anthrax disease	1	Fruit shape	44
Resistance to Potato Y Virus	4	Fruit weight	49
Resistance to bacterial wilt	30	Number of ventricles	14
Resistance to thrips	4	Single node with multi flower	177
Chlorophyll	73	Corolla color	55
Inhibition α - glucosidase	4	Peduncle length	45
Dry matter content	44	Anther color	78
Capsaicin	17	The node position of flowers	167
Flavonoid	10	GMS	5
Purple fruit	48	Male sterile	2
Fruit flavor	23	Branch type	304
VC content	7	Main stem color	22
Fruit color	16	Plant architecture	124
Anthocyanin	2	Pigmented peppers	8
Leaf surface fuzziness	6	Stem hairiness	11
Leaf margin	78		

### Verification of the genotyping performance of the gene chip

The performance of the gene chip was evaluated through genotyping of 420 diverse pepper germplasms (**Supplementary Table S1**). The pepper genomic DNA library fragments were sized at approximately 300 bp, and the sequencing of all materials yielded more than 630 Gb of raw data, with each accession yielding at least 1.5 Gb. This translates to an average sequencing depth of about 112x for each core polymorphic site. Among the 45,389 probes, 45,387 successfully captured target DNA fragments. The average capture efficiency of the probes for each pepper DNA sample was 97.72%, with 93.2% of the pepper DNA samples displaying a capture efficiency of 98.04% or higher (**Figure 1E**). After sequencing the captured fragments, a total of 117,275 SNPs and 8,902 small indels were identified. The polymorphic sites with a detection ratio of over 95% accounted for 94.2% of all sites among the 420 pepper samples. Based on the capture efficiency, 410 samples (with a probe capture ratio of ≥70%) were retained for further analyses. All foreground probes were successful in capturing fragments in these 410 pepper germplasms, with an average capture ratio of 99.83%. The low detection rates in 10 samples may be attributed to the variable DNA quality and integrity, as DNA extraction was not performed in a single batch. Although the DNA from some materials may meet the minimum requirements for subsequent experiments, the outcomes may not be optimal. Moreover, incomplete reactions during any step of DNA library construction, hybridization with the probes, elution of the bound fragments, or sequencing could also contribute to reduced detection rates.

To assess the application of foreground probes in genotyping pepper germplasms for spiciness traits, we categorized the spiciness of fruits into “sweet,” “mildly spicy,” “moderately spicy,” and “spicy” based on taste. Among the 273 germplasms with evaluated spiciness, there were 31 “sweet,” 4 “mildly spicy,” 140 “moderately spicy,” and 97 “spicy” germplasms (**Supplementary Table S1**). Sequencing data for 15 spiciness-associated foreground probes were subsequently extracted from these 273 germplasms. All 15 spiciness-related probes captured fragments, with 4 probes detecting 2-3 SNPs each (**Table 2**). Furthermore, a specific nucleobase pattern labeled as “6 CPS” was found exclusively in “sweet” pepper germplasms (**Table 2**). Both Spicy6 and Spicy7 probes showed consistent genotyping results across all spiciness categories, and probes located on chromosome 12 (Spicy9 - Spicy15) yielded identical genotyping results, suggesting that segments on chromosomes 10 and 12 are inherited as a block between different generations (**Table 2**).

**Table 2:** Genotypic characterization of pepper germplasms with different spiciness types.

Probe	Position	Sweet	Sweet spicy	Mild spicy	Spicy
Spicy1	Chr1:205543375	0.00% (A)	41.18% (A)	38.95% (A)	72.41% (A)
Spicy2	Chr1:308205698	35.71% (A)	17.65% (A)	85.26% (A)	29.31% (A)
Spicy3	Chr4:64371854	0.00% (G)	11.76% (G)	32.63% (G)	31.03% (G)
Spicy4	Chr6:36083447	0.00% (G)	11.76% (G)	3.16% (G)	27.59% (G)
Spicy5	Chr9:209508382	0.00% (A)	17.65% (A)	12.63% (A)	5.17% (A)
Spicy6	Chr10:106247954	0.00% (G)	17.65% (G)	25.26% (G)	34.48% (G)
Spicy7	Chr10:149802540	0.00% (A)	17.65% (A)	25.26% (A)	34.48% (A)
Spicy8	Chr10:37668892	7.14% (T)	17.65% (T)	33.68% (T)	68.97% (T)
Spicy9	Chr12:38386021	14.29% (G)	41.18% (G)	81.05% (G)	93.10% (G)
Spicy9	Chr12:38386172	14.29% (C)	41.18% (C)	81.05% (C)	93.10% (C)
Spicy9	Chr12:38386176	14.29% (C)	41.18% (C)	81.05% (C)	96.55% (C)
Spicy10	Chr12:38428906	14.29% (A)	41.18% (A)	81.05% (A)	96.55% (A)
Spicy10	Chr12:38428922	14.29% (G)	41.18% (G)	81.05% (G)	96.55% (G)
Spicy11	Chr12:38493816	14.29% (C)	41.18% (C)	81.05% (C)	96.55% (C)
Spicy12	Chr12:38504365	14.29% (C)	41.18% (C)	81.05% (C)	96.55% (C)
Spicy13	Chr12:38510398	14.29% (T)	41.18% (T)	81.05% (T)	96.55% (T)
Spicy13	Chr12:38510606	14.29% (T)	41.18% (T)	81.05% (T)	96.55% (T)
Spicy14	Chr12:38511909	14.29% (G)	41.18% (G)	81.05% (G)	96.55% (G)
Spicy15	Chr12:38744126	14.29% (C)	41.18% (C)	81.05% (C)	96.55% (C)
Spicy15	Chr12:38744168	14.29% (G)	41.18% (G)	81.05% (G)	96.55% (G)

### Evolutionary and phylogenetic analysis of pepper germplasms

**Figure 2:**
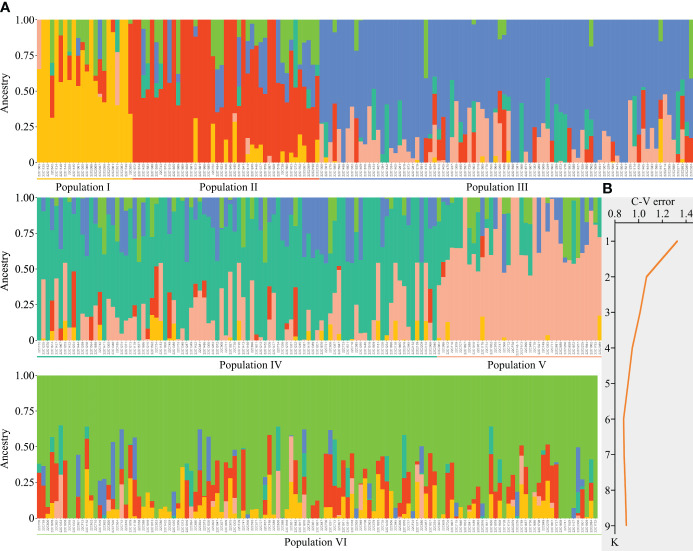
Population structure analysis of 410 pepper germplasms. **(A)** The 410 pepper germplasms can be classified into six distinct populations. **(B)** Cross-validation (CV) error assessment for the K value.

Through population structure analysis and iterations assessing cross-validation error (CV error) values, a pronounced demarcation was manifest when setting the CV error value to 6, demonstrating significant differentiation with the minimal error value. Consequently, it was preliminarily deduced that the 410 pepper samples could be taxonomically stratified into 6 distinct populations (**Figure 2**). Population I predominantly encompassed 21 accessions, unique for their goat horn-shaped, helical fruits; the bulk of *C. chinense* samples were grouped into Population II; Populations III and IV almost exclusively comprised various cultivars of skyward-pointing peppers; and finally, Populations V and VI were characterized mainly by lantern-shaped and sweet pepper varieties, respectively (**Figure 2**; **Supplementary Table S1**). Furthermore, accessions with high pungency and those devoid of spiciness were seldom found within the same population (**Figure 2**; **Supplementary Table S1**), signifying the gene chip’s adeptness at discerning individuals across different populations.

**Figure 3:**
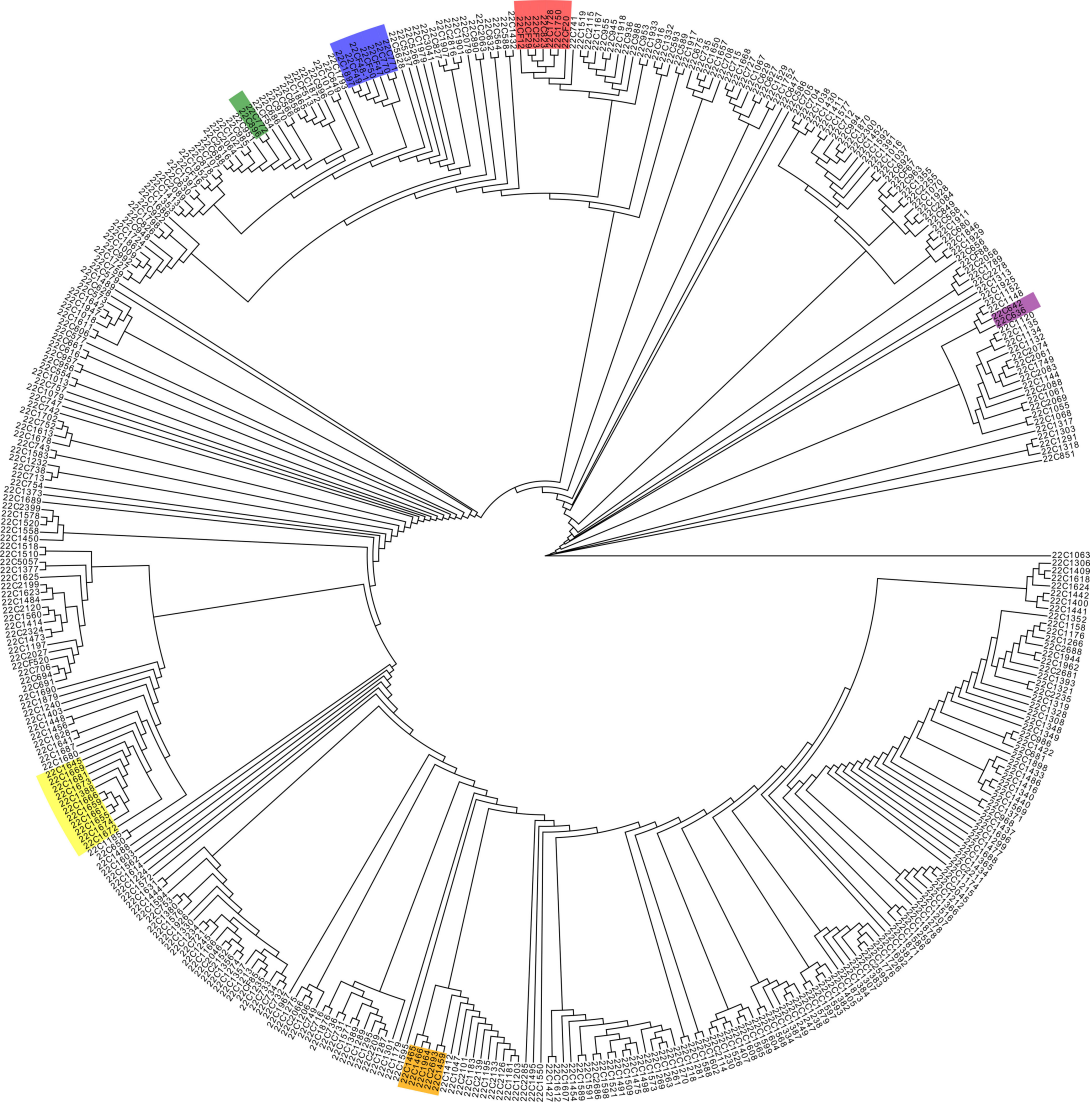
IQtree analysis of the 410 pepper germplasms. The color lumps in the tree diagram indicate sister lines or *Capsicum chinense*, identified as neighboring branches.

In addition, gene chip sequencing data facilitated the conduct of an IQ-TREE analysis of the 410 pepper accessions (**Figure 3**). This analysis showed that accessions with congruent genetic backgrounds tended to cluster together. Notably, sister lines such as 22C1458, 22C1459, 22C1466, 22C1964, and 22C1465 were grouped on the same phylogenetic branch; a substantial number of *C. chinense* samples, including 22C1645, 22C1669, 22C1681, 22C1673, 22C1666, 22C1659, 22C1661, 22C1655, 22C1674, and 22C1672, formed a cohesive group, with sister lines 22C1655, 22C1659, and 22C1661 clustering into a narrower branch; the self-pollinated lines derived from backcrosses involving 22C823, which carries the L4 gene, including 22CF12, 22CF29, 22CF23, and 22CF20, were clustered into a single group; and accessions 22C1897, 22CF49, 22CF48-1, and 22CF50 were classified in the same branch, those accessions were all backcross progenies of 22CF47 which carried *msc-1* gene and beared long horn-shaped mild spiciness fruits (**Figure 3**). Moreover, 22C772, a self-pollinated line derived from the cross between 22CF47 and 22C896, was positioned alongside 22C896 in the smallest division of the IQ-TREE (**Figure 3**), affirming the chip’s efficacy in IQ-TREE analysis. Furthermore, peppers with high levels of spiciness were situated on distinct branches separate from those with low spiciness, while sweet peppers were grouped in the same major branch alongside mildly spicy peppers (**Figure 3**). This suggests that the 45K liquid-phase pepper gene chip is capable of effectively distinguishing between peppers with similar genetic backgrounds.

**Figure 4:**
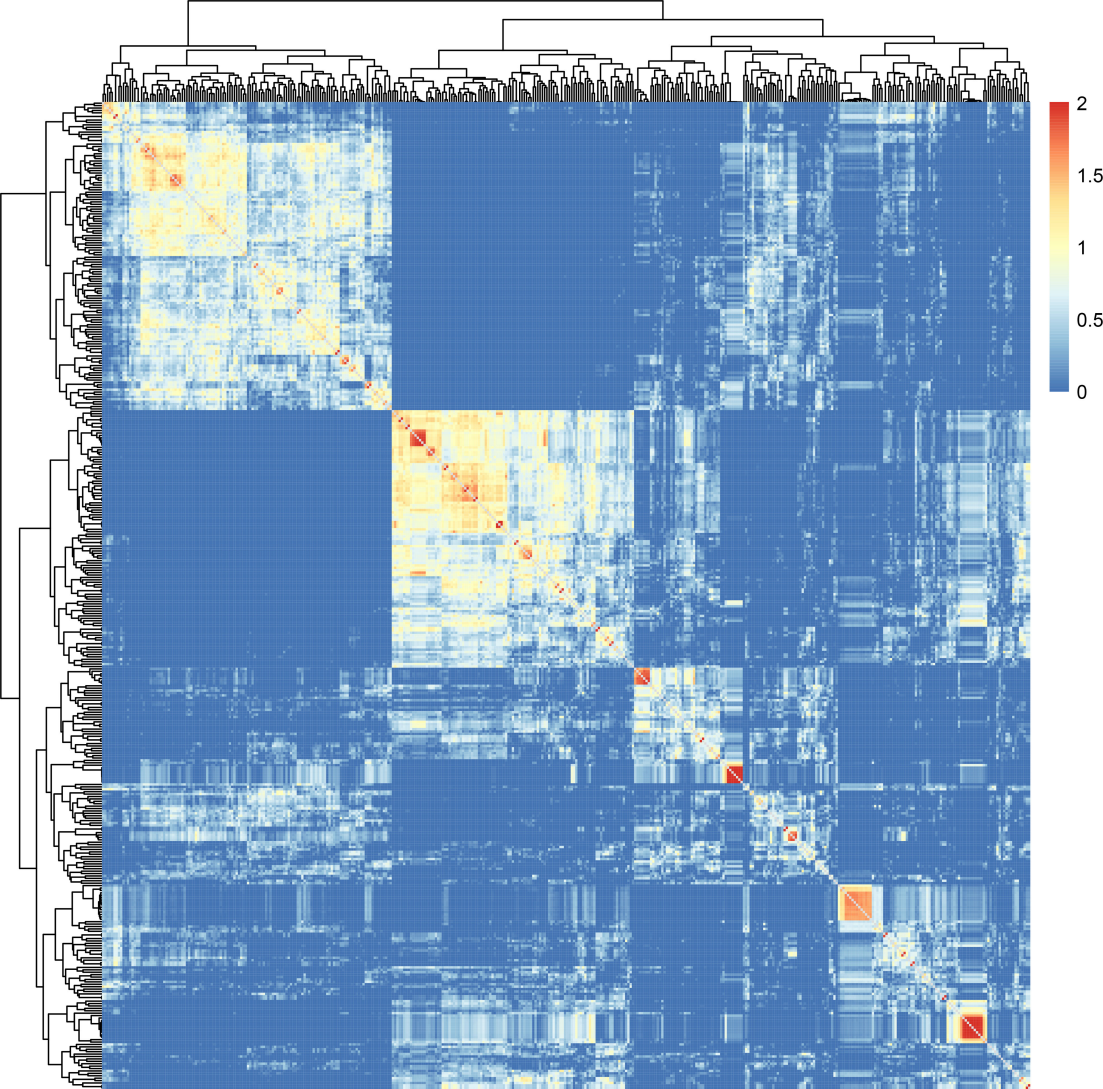
Genetic correlation matrix of the pepper germplasms.

Lastly, Finally, kinship analysis conducted on the 410 pepper germplasms using TASSEL software revealed that the majority (83.26%) of accessions had kinship values between 0 and 0.5, with only 5.92% of accessions having kinship values exceeding 1.0. The average kinship value was 0.29, indicating that most germplasms originated from different families, while a portion consisted of closely related sister lines or inbred lines (**Figure 4**). This result is consistent with the actual situations of these pepper germplasms (**Figure 4**).

### GWAS of the helical-shaped fruit trait in pepper germplasms

**Figure 5:**
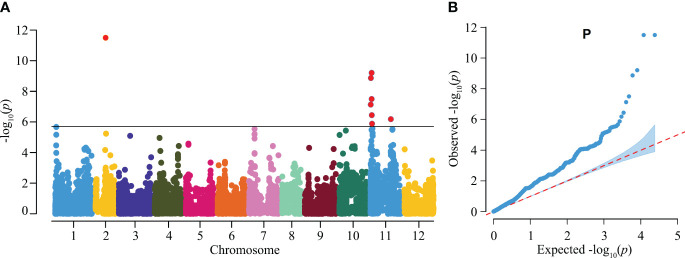
Genome-wide association study (GWAS) of the helical-shaped fruit morphology in pepper germplasms. **(A)** Manhattan plot illustrating the GWAS analysis for the helical-shaped fruit trait in pepper germplasms. **(B)** Quantile-quantile (Q-Q) plot for the GWAS evaluation.

To further demonstrate the applicability of the gene chip in GWAS, we focused on the trait of the helical-shaped fruit, a unique agronomic trait with significant market appeal in certain regions of China. However, the loci and genes regulating this trait are not well-documented. In this study, we utilized the gene chip sequencing data as well as the helical-shaped fruit observation data from 384 germplasms for GWAS. Among these samples, 66 exhibited the helical-shaped fruit phenotype, including helical-shaped horn and helical-shaped bullhorn peppers (**Supplementary Table S1**). The GWAS identified 3 significant QTLs on chromosomes 2 and 11, named *heli2.1*, *heli11.1*, and *heli11.2* (**Figure 5**), containing a total of 9 SNPs significantly associated with the trait, with phenotypic variance explanation rates ranging from 6.64% to 12.53% (**Table 3**). Seven of these SNPs were located in intergenic regions, not within potential promoter regions (3 kb upstream of start codons), and the remaining two SNPs were located in the exons of Chr11g001780 and Chr11g002450 (**Table 3**).

**Table 3:** QTLs controlling helical-shaped fruits in pepper.

QTLs	Marker and Position	Gene ID	*P*-value	Contribution rate
*heli2.1*	Chr02_90,049,878	Between Chr02g007170 and Chr02g007180	3.17E-12	0.12353
*heli2.1*	Chr02_90,049,913	Between Chr02g007170 and Chr02g007180	3.17E-12	0.12353
*heli11.1*	Chr11_3,585,223	Chr11g001780 exon	7.37E-08	0.07964
*heli11.1*	Chr11_4,841,180	Chr11g002450 exon	1.36E-09	0.09759
*heli11.1*	Chr11_11,717,741	Between Chr11g005030 and Chr11g005040	6.19E-10	0.10107
*heli11.1*	Chr11_11,749,401	Between Chr11g005040 and Chr11g005050	3.18E-08	0.08358
*heli11.1*	Chr11_13,044,490	Between Chr11g005370 and Chr11g005380	3.61E-07	0.07236
*heli11.1*	Chr11_15,115,808	Between Chr11g005900 and Chr11g005910	1.31E-06	0.06638
*heli11.2*	Chr11_183,571,838	Between Chr11g015600 and Chr11g015610	6.57E-07	0.0721

To identify potential candidate genes for controlling the helical-shaped fruit trait, genes within a 2 Mb region upstream and downstream of the significantly associated SNPs were extracted. In the *heli2.1*, 25 annotated genes (Chr02g007090 to Chr02g007330) were identified, including genes encoding LRR receptor-like, PP2-like, and RLP12, etc. (**Supplementary Table S3**). The *heli11.1* locus contained 63 genes (Chr11g004640 to Chr11g005260), with notable genes such as genes encoding Aquaporin NIP, NAC domain-containing, and AD-linked sulfhydryl oxidase (**Supplementary Table S3**). In the *heli11.2* locus, 10 genes were identified (Chr11g015530 to Chr11g015620), including the genes encoding ABC transporter, MTM1, and MIB1 etc. (**Supplementary Table S3**).

## Discussions

### Advantages and disadvantages of the 45K pepper GBTS liquid-phase gene chip

The chip developed in this study comprised 45,389 probes, aligning with the mainstream level of GBTS chips utilized in crops such as maize, soybean, and cotton, and substantially surpassing the probe count of the three extant pepper gene chips (the 30K solid-phase, 15K solid-phase, and 5K liquid-phase) (Hill et al., 2013; Cheng et al., 2016; Liu et al., 2020; Guo et al., 2021; Si et al., 2022; Miao et al., 2023; Yang et al., 2023). However, in terms of the density of core polymorphic sites, since the pepper genome (~3.0 Gb) was much larger than those of maize (~2.3 Gb), soybean (~1.0 Gb), and cotton (~2.2 Gb), the density of core polymorphic sites in this chip was about 14.88/Mb, which is less than that of the soybean 40K chip (41.54/Mb), maize 40K chip (17.39/Mb), and cotton 40K chip (18.12/Mb) (Liu et al., 2020; Guo et al., 2021; Si et al., 2022; Yang et al., 2023). With regards to the uniformity of probe distribution, this chip differed from the soybean 40K chip (Yang et al., 2023) as it did not reveal a notable variance in distribution density between euchromatin and heterochromatin regions on chromosomes (**Figure 1B**), mirroring the distribution in GBTS chips developed for soybean, maize, and cotton (Liu et al., 2020; Guo et al., 2021; Si et al., 2022).

On the aspect of foreground probes, the number and quality of foreground sites/probes depended on the existing research foundation of the very crop. In the recently reported wheat GBTS gene chip, its probes were developed based on 101 functional or closely linked markers published over the past 22 years, covering 13 agronomic traits including stripe rust, powdery mildew, pre-harvest sprouting, and grain weight (Xiang et al., 2023). Similarly, in this study, referencing 89 publications over the past 24 years, we developed 1,869 molecular markers related to disease resistance, important secondary metabolites, and plant and fruit morphology (**Table 1**). By genotyping for spiciness type, the accuracy of these associated polymorphic sites was demonstrated, laying the foundation for the future development of a miniaturized foreground site pepper gene chip. However, it is worth noting that since the information on foreground sits depends on previous researches, limited by the number and variety of pepper germplasms tested in this study, the accuracy of these foreground sites and their ability to inspect different alleles were limited, and this situation also existed in other gene chips as well (Xiang et al., 2023). Thanks to the advantage of being able to add probes to the liquid-phase gene chip at any time, as the QTLs controlling different agronomic traits in peppers is continuously explored, the range and accuracy of the foreground sites in this 45K pepper GBTS liquid-phase gene chip will be continually expanded and improved.

### Genome-wide association study of the helical-shaped fruit trait

To further verify the applicability of the gene chip in GWAS, we performed GWAS of helical-shaped fruit trait in our collection of pepper germplasms. Finally, three QTLs controlling helical shape of pepper fruit were identified on chromosomes 2 and 11 (**Figure 5**; **Table 3**; **Supplementary Table S3**). This result is consistent with the observation during the long-term breeding, that the helical shape of pepper fruit is a quantitative trait, since the rotation angle the helical-shaped fruits in the F_2_ segregation population continuously changed. As to the identification of the candidate genes controlling helical-shaped fruit, we discovered a gene encoding tubulin alpha chain (Chr11g004680) was located in heli11.1 (**Supplementary Table S3**). Alpha-tubulin has been confirmed to be involved in the regulation of spiral phenotypes. For example, the *tid1-1* mutant in rice, caused by a mutation where the 56^th^ amino acid of alpha-tubulin changes from threonine to isoleucine, leads to right-spiral growth of leaves and stems (Sunohara et al., 2009); in Arabidopsis, mutations in the *LEFTY* gene, which is highly homologous to *TID*, result in left-spiral growth of the roots which is in the opposite direction to the rice *tid1-1* mutant; the Arabidopsis right-spiral growth mutant *tortifolia2* is also caused by a mutation in alpha-tubulin (Buschmann et al., 2019). Therefore, Chr11g004680 was an important candidate gene for helical-shaped fruit in pepper. However, it is worth noting that there were still 19 genes with unknown function within the three QTLs, and whether there were genes regulating helical shape of fruit remained to be validated by more future researches.

In summary, the 45K pepper GBTS liquid-phase gene chip developed in this study provided a powerful tool for pepper breeding, characterized by a large number of probes, high efficiency in polymorphic sites detection, even genomic distribution, and broad applications in germplasm identification, phylogenetic analysis, and agronomic trait locus discovery. Therefore, this gene chip has enormous potential for application in future breeding and germplasm research in pepper.

## Data availability statement

The original contributions presented in the study are included in the article/**Supplementary Material**, further inquiries can be directed to the corresponding author/s.

## Author contributions

ZL: Data curation, Formal analysis, Methodology, Software, Validation, Visualization, Writing – original draft, Writing – review & editing. ZJ: Formal Analysis, Methodology, Validation, Writing – original draft. JL: Conceptualization, Funding acquisition, Project administration, Writing – original draft. DK: Conceptualization, Funding acquisition, Project administration, Writing – original draft. ML: Formal analysis, Writing – original draft. SM: Formal analysis, Writing – original draft. QC: Project administration, Writing – review & editing. LS: Conceptualization, Formal analysis, Funding acquisition, Investigation, Project administration, Resources, Supervision, Writing – original draft, Writing – review & editing. HS: Conceptualization, Formal analysis, Funding acquisition, Investigation, Project administration, Resources, Supervision, Writing – original draft, Writing – review & editing.
